# Characterization of *Mycobacterium tuberculosis *Central Asian Strain1 using mycobacterial interspersed repetitive unit genotyping

**DOI:** 10.1186/1471-2180-7-76

**Published:** 2007-08-09

**Authors:** Asho Ali, Zahra Hasan, Mahnaz Tanveer, Amna R Siddiqui, Solomon Ghebremichael, Gunilla Kallenius, Rumina Hasan

**Affiliations:** 1 Department of Pathology and Microbiology, The Aga Khan University, Stadium Road, PO Box 3500, Karachi, Pakistan; 2 Department of Community Health Sciences, The Aga Khan University, Stadium Road, PO Box 3500, Karachi, Pakistan; 3 Department of Bacteriology, Swedish Institute for Infectious Diseases Control, Stockholm, Sweden; 4 Microbiology and Tumor Cell Biology, Karolinska Institute, Nobels Vag 16, Stockholm, Sweden

## Abstract

**Background:**

The Central Asian Strain1 (CAS1) genogroup of *Mycobacterium tuberculosis *(MTB) is the most prevalent in Pakistan, India and Bangladesh. Mycobacterial interspersed repetitive units variable number tandem repeat (MIRU-VNTR) typing is a reliable and reproducible method for differentiation of MTB isolates. However, information of its utility in determining the diversity of CAS1 strain is limited. We performed standard 12 loci based MIRU-VNTR typing on previously spoligotyped CAS1 strains and 'unique' strains in order to evaluate its discriminatory power for these isolates.

**Methods:**

Twelve loci based MIRU- VNTR typing was used to type178 CAS1 and 189 'unique' MTB strains. The discriminatory index for each of the loci was calculated using the Hunter Gaston Discriminatory Index (HGDI). A subset of these strains (n = 78) were typed using IS*6110 *restriction fragment length polymorphism (RFLP). MIRU-VNTR profiles were studied together with their drug susceptibility patterns.

**Results:**

A total of 349 MIRU patterns were obtained for the 367 strains tested. The CAS1 strains were subdivided into 160 distinct patterns; 15 clusters of 2 strains each, 1 cluster of four strains and 144 unique patterns. Using HGDI, seven MIRU loci, (numbers 26, 31, 27, 16, 10, 39, and 40) were found to be "highly discriminatory" (DI: ≥0.6), four MIRU loci (numbers 20, 24, 23, and 4) were "moderately discriminatory" (DI: 0.3–0.59), and one locus (number 2) was "poorly discriminatory" (DI< 0.3). Loci 26 and 31 were the most discriminatory for the CAS1 isolates. Amongst 'unique' strains in addition to loci 26, 31, 27, 16, 10, 39, and 40, locus 23 was highly discriminatory, while no locus was poorly discriminating. DI values for loci 4, 10 and 26 were significantly lower (P-value < .01) in CAS1 strains than in 'unique' strains. The association between CAS1 strains and MDR was not found to be significant (p value = 0.21).

**Conclusion:**

We propose that MIRU typing could be used to estimate the phylogenetic relatedness amongst prevalent CAS1 strains, for which MIRU loci 26, 31, 16, 10, 27, 39 and 40 were found to be the most discriminatory.

## Background

Pakistan, together with other Asian countries including; China, India, Bangladesh, and Indonesia, shares over 50 percent of the global burden of the tuberculosis (TB) cases [1,2]. Pakistan ranks sixth amongst the 22 high burden TB disease countries [1], with an estimated incidence rate of 171/100,000 population. Despite this the TB burden is an underestimated figure as many cases in the country go unreported due to lack of access to health care facility, over crowding, poverty and other social constraints.

The high incidence of tuberculosis in Pakistan is further compounded by the increasing emergence of drug resistant strains including multi-drug resistant (MDR: resistant to at least Rifampicin and Isoniazid) strains. The global prevalence of MDR is estimated at 3% [3-5]. However China, Iran and India report MDR-TB at 4.5%, 5% and 3.4% respectively [5]. While community based data from Pakistan is currently not available, laboratory based studies from urban Rawalpindi showed an increasing frequency of MDR from 14% in 1999 to 28% in 2004 [6] and a study from a tertiary care center in Karachi documented 47% MDR-TB prevalence [7].

Key factors required for effective control of TB are rapid detection, adequate therapy and a better understanding of TB epidemiology to understand the transmission patterns of the disease. *Mycobacterium tuberculosis *(MTB), the main causative agent of TB has an overall genomic similarity of 99.9% [8,9]. There is moreover increasing evidence that specific genetic differences within MTB may be associated with geographical locations [10-14]. Thus studies of the genetic diversity of MTB in a high burden country such as Pakistan are required in order to provide insight into dissemination dynamics and virulence pattern of the pathogen.

Genotyping methods such as PCR based spacer oligonucleotide typing (spoligotyping) have facilitated differentiation of MTB isolates into predominant genogroups including, the Beijing family of strains and Central Asian Strain1 (CAS1). We have reported CAS1 strains lacking spacers 4–7 and 23–34 to be the most prevalent (39%) in Pakistan [15]. CAS1 has also been reported as the second most predominant group in South Asia; India (16–22%) and Bangladesh (17%) [15-18]. Whilst, Beijing strains lacking spacers 1–34 are the most widely reported genotype world wide [19,20] and the most prevalent genotype in East Asia and Russia (40–60%) [21-24], they constitute only 6% of MTB isolates in Pakistan [15]. Despite the predominance of CAS1 in South Asia, there is limited data related to its transmission and drug resistance [25]. Spoligotyping while instrumental in identifying MTB genogroups is unable to help discriminate amongst them. Mycobacterial interspersed repetitive units variable number tandem repeat (MIRU-VNTR) is based on detection of independent mini satellite like loci scattered through out the MTB genome and has been shown to be a reliable and reproducible typing method with high discriminatory power [26-29] for studying the MTB population structure in different countries [28,30]. The typed strains are expressed by a 12-digit numerical code, corresponding to the number of repeats at each locus [31,32]. This numerical code is easy to compare and exchange at inter-, and intra-laboratory level. The discriminatory power of MIRU-VNTR analysis is proportional to the number of loci evaluated. In general, the discriminatory power of standard twelve loci based typing only slightly lower than that of the IS*6110 *based restriction fragment length polymorphism (RFLP), which is currently the gold standard for MTB genotyping [29].

Twelve loci based MIRU-VNTR analysis has been used in a number of molecular epidemiologic studies and to elucidate the phylogenetic relationship of clinical isolates [28,30,33-35]. It has also been used to study Beijing strains from East and South Asia [27,36-40]. Available data for MIRU-VNTR typing for MTB in Pakistan is limited to one report wherein five exact tandem repeat (ETR) were used to type 113 MTB isolates from Rawalpindi, Pakistan. This showed clustering of one third of the isolates, which were further discriminated by an IS*6110 *based analysis [25].

In this study we have used standard 12 MIRU-VNTR loci typing to identify the alleles most discriminatory for CAS1 as compared with 'unique' spoligotypes within MTB strains selected from different geographical location in Pakistan. We have also determined the association of these strains with MDR.

## Results

### MIRU typing for the predominant CAS1 genogroup and 'unique' strains from Pakistan

**Figure 1:**
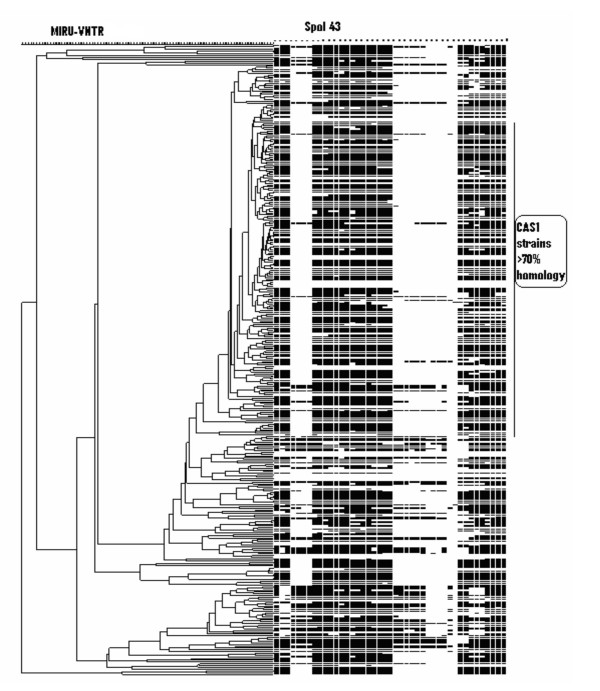
**MIRU-VNTR typing of *Mycobacterium tuberculosis *from Pakistan**. Three hundred and sixty seven strains were typed and a cluster analysis was carried out using Bionumerics software using the unweighted pair group method. The 178 CAS1 strains studied showed an overall homology of >70%. No MIRU clusters were observed between any of the 189 'unique' strains studied.

The twelve loci MIRU-VNTR analysis detected a total of 349 MIRU patterns in our sample size of 367 strains (Fig 1). The 178 strains of the CAS1 genogroup were found to be more than 70 % homologous, but were further divided into 160 distinct patterns comprising of; 15 clusters of two strains each, 1 cluster of four strains and with 144 non-matching patterns. The 189 strains previously identified by spoligotyping as 'unique' [15] remained unclustered after MIRU analysis. The distribution of the MIRU alleles is summarized in Table 1.

**Table 1:** Allelic diversity of 367 *Mycobacterium tuberculosis *isolates from Pakistan.

**MIRU **number	**Allele Number**										**Allelic Diversity**	**Rank**	**Conclusion**
				
	**0**	**1**	**2**	**3**	**4**	**5**	**6**	**7**	**8**	**9**			
**2**	18	85	262	2							0.4355	11	*Moderately discriminant
**4**	21	13	290	9	11	22	1				0.3670	12	Moderately discriminant
**10**	18	4	29	48	73	133	50	8	4		0.7862	3	#Highly discriminant
**16**	43	19	46	126	83	40	9		1		0.7885	2	Highly discriminant
**20**	22	60	248	29	3	4			1		0.5080	10	Moderately discriminant
**23**	11	4	4	10	49	234	42	8	4	1	0.5616	8	Moderately discriminant
**24**	77	238	34	2	3	12		1			0.5271	9	Moderately discriminant
**26**	9	24	20	12	31	58	96	84	30	3	0.8337	1	Highly discriminant
**27**	20	23	107	167	42	8					0.6893	7	Highly discriminant
**31**	18	4	30	101	116	73	25				0.7731	4	Highly discriminant
**39**	22	60	153	116	15	1					0.6962	6	Highly discriminant
**40**	11	31	116	150	51	6	2				0.7073	5	Highly discriminant

	Average										0.6394		

Discriminatory Index:^#^≥0.6 = Highly Discriminant and *0.3–0.59 = Moderately Discriminant

### Allelic diversity

Allelic diversity of clinical isolates was determined by twelve MIRU loci analysis using the Hunter Gaston Discriminatory Index (HGDI). Overall, MIRU-VNTR typing of 367 MTB strains indicated a discriminatory power of 0.999. Diversity of CAS1 (n: 178) and 'unique' (n: 189) strains was further calculated separately (Table 1 and 2). Allelic analysis of 178 CAS1 strains showed a HGDI of 0.998.

**Table 2:** Twelve MIRU loci analysis of CAS1 and 'unique' spoligotypes

**MIRU Loci**	**HGDI values for**				**P- value**
		
	**CAS1 Strains (n = 178)**	**Conclusion**	**'unique' Strains (n = 189)**	**Conclusion**	
2	0.4953	Moderately discriminant	0.3859	Moderately discriminant	0.105
4	0.2676	Poorly discriminant	0.4540	Moderately discriminant	0.000*
10	0.7449	Highly discriminant	0.8190	Highly discriminant	0.004*
16	0.7503	Highly discriminant	0.7760	Highly discriminant	0.325
20	0.5993	Moderately discriminant	0.4242	Moderately discriminant	0.124
23	0.5211	Moderately discriminant	0.6099	Highly discriminant	0.126
24	0.5709	Moderately discriminant	0.4966	Moderately discriminant	0.436
26	0.8117	Highly discriminant	0.8511	Highly discriminant	0.000*
27	0.7588	Highly discriminant	0.6151	Highly discriminant	0.909
31	0.7772	Highly discriminant	0.7756	Highly discriminant	0.340
39	0.7090	Highly discriminant	0.6775	Highly discriminant	0.732
40	0.6970	Highly discriminant	0.7228	Highly discriminant	0.661

	Average: 0.6419		Average: 0.6339		

Significantly different loci are indicated by '*' (P value < 0.05)

Allelic diversity for each locus was calculated in order to determine the discriminatory power of these loci in a combined group for the MTB population studied. Overall, the average allelic diversity of loci studied in these strains was found to be 0.6394 (Table 1). Based on their discriminatory index (DI), seven MIRU loci 10, 16, 26, 27, 31, 39 and 40 were designated as "highly discriminant" (DI ≥ 0.6). While, MIRU loci 2, 4, 20, 23 and 24 were designated as "moderately discriminant" (0.3 ≤ DI ≤ 0.6) [28]. In our MTB population locus 26 was found to be the most discriminatory allele in order to distinguish between CAS1 strains and 'unique' spoligotypes. Locus 26 provided a 10 allelic discrimination with a HGDI of 0.833. This was followed by loci 16, 10, 31, 40, 39 and 27 respectively in order of decreasing discrimination. Locus 4 was found to be the least discriminatory with 7 alleles and a HGDI of 0.367.

As shown in Table 2, the average allelic diversity of CAS1 strains was found to be 0.6419. Of these, seven MIRU loci, numbers 26, 31, 27, 16, 10, 39, and 40, were "highly discriminant" (DI: ≥ 0.6); four MIRU loci, 20, 24, 23, and 4 were "moderately discriminant" (DI: 0.3–0.59); while locus number 4 was "poorly discriminant" (DI< 0.3) for CAS1 isolates.

The average allelic diversity of 'unique' strains was found to be 0.6339 (Table 2). The diversity patterns observed for 'unique' strains was similar that found for CAS1 strains, i.e. eight MIRU loci, number 26, 10, 16,31, 40, 39, 27 and 23 were "highly discriminant" (DI:≥0.6) and four loci numbers 24, 4, 20 and 2 were "moderately discriminant" (DI:0.3–0.59). However, no loci for 'unique' strains were identified to be "poorly discriminant".

### Discriminatory power of MIRU-VNTR typing for CAS1

Further statistical analysis was carried out to investigate the utility of each of the twelve loci of MIRU typing to distinguish between CAS1 and 'unique' strains. Data was analyzed using the non-parametric Mann-Whitney test. Results revealed that differences in loci 4, 10 and 26 were statistically significant (P-value < .01).

### IS*6110*-RFLP typing

**Figure 2:**
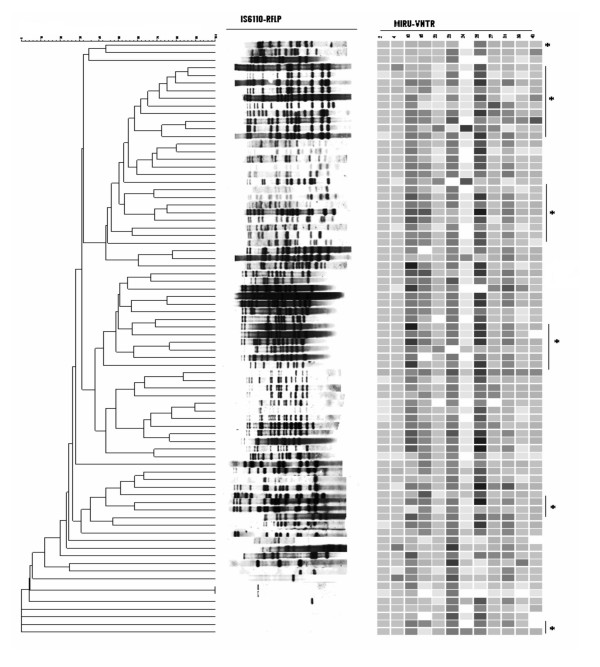
**IS*6110*-RFLP typing of *Mycobacterium tuberculosis***. The figure illustrates a composite analysis of IS*6110*-RFLP and MIRU-VNTR of 78 MTB strains using Bionumerics software (Applied Maths). The strains included 29 CAS1 and 49 'unique' strains. * denotes CAS1 strains exhibiting heterogeneous IS*6110*-RFLP profiles.

To further investigate the heterogeneous pattern shown by MIRU-VNTR typing, IS*6110*-RFLP typing was carried on a subset of strains; 29 CAS1 and 49 'unique' spoligotypes. IS*6110*-RFLP typing of these 78 strains resulted in 73 different RFLP types (Fig 2). One cluster of two strains, with single copy of IS*6110 *was identified, which was further discriminated into individual patterns by MIRU-VNTR typing. The remaining seventy two strains revealed unique RFLP patterns while four strains were of 'zero' copy IS*6110*. Despite the heterogeneous fingerprint pattern shown by RFLP based clustering, the 25 CAS1 strains with multiple IS*6110 *copy exhibited 60% homology. About one fourth of the strains tested had 13 copies of IS*6110 *element.

### Comparison of MDR isolates

We analyzed MIRU patterns for all the MDR strains in order to investigate an association between resistance and MIRUs. Of the CAS1 strains studied, 62 were MDR (53%) while 54 'unique' strains were MDR (47%). HGDI values of MIRU loci in MDR strains are shown in Table 3. Locus 4 was found to be statistically significant in discriminating between CAS1 and 'unique' MDR strains. Overall, no significant difference could be established between MIRU patterns of CAS1 and 'unique' MDR isolates.

**Table 3:** MIRU loci analysis of MDR *M tuberculosis*

**MIRU Loci**	**HGDI values for**				**P- value**
		
	**MDR CAS1 (n = 62)**	**Conclusion**	**MDR 'unique' (n = 54)**	**Conclusion**	
2	0.5944	Moderately discriminant	0.4983	Moderately discriminant	0.463
4	0.2871	Poorly discriminant	0.4689	Moderately discriminant	0.007*
10	0.7536	Highly discriminant	0.8092	Highly discriminant	0.142
16	0.7784	Highly discriminant	0.7582	Highly discriminant	0.188
20	0.6076	Highly discriminant	0.3662	Moderately discriminant	0.098
23	0.5463	Moderately discriminant	0.5311	Moderately discriminant	0.862
24	0.5219	Moderately discriminant	0.4780	Moderately discriminant	0.266
26	0.8080	Highly discriminant	0.8609	Highly discriminant	0.100
27	0.7504	Highly discriminant	0.5542	Moderately discriminant	0.955
31	0.7583	Highly discriminant	0.7659	Highly discriminant	0.934
39	0.7155	Highly discriminant	0.6003	Highly discriminant	0.465
40	0.7150	Highly discriminant	0.7358	Highly discriminant	0.332

	Average: 0.6530		Average: 0.6189		

*Locus 4 between the two is significantly different (P value < 0.05)

## Discussion

We have used MIRU-VNTR typing to characterize the predominant CAS1 genogroup of *Mycobacterium tuberculosis *strains in Pakistan. Limited information is available about the utility of MIRU-VNTR typing method to characterize CAS1 strains i.e. 2–24 [18,41,42]. This study presents the largest MIRU-VNTR genotyping data for Pakistani isolates to date. Using 12 loci based MIRU-VNTR typing we studied a population of 367 MTB strains and found them to be highly diverse. Of the 178 CAS1 strains studied only 34 (19 %) clustered into groups based on MIRU profiles, while all 189 'unique' spoligotypes studied had non-matching MIRU profiles and therefore remained unclustered.

Twelve loci based MIRU-VNTR typing has been extensively used to study Beijing strains. Results of these studies indicates Beijing strains to display variable clustering, between 53–100% [18,33,37,43,44]. Amongst the Beijing isolates, locus 10 has been found to be the most discriminatory followed by locus 26 and 31, while other loci being almost monomorphic [37,44]. In contrast, using 12 loci based MIRU-VNTR analysis we were unable to identify any monomorphic loci within the CAS1 genogroup. Our data showed MIRU loci 26, 31, 16, 10, 27, 39 and 40, in decreasing order, to be the most discriminatory for the CAS1 genogroup of *Mycobacterium tuberculosis*. Despite exhibiting genetic phylogenetic variability CAS1 strains studied also revealed more than 70% homology in their MIRU profile. This could either be due to an intrinsic similarity within the CAS1 genogroup or may be reflective of relatedness between strains found in a particular geographical region.

The overall allelic diversity and discriminatory power of the VNTR loci in the MTB isolates of CAS1 and ' unique' spoligotypes studied from Pakistan were higher than that reported earlier for strains from Singapore, Russia and South Africa [37,42]. The greater diversity observed can be attributed to continual import of new strains due to traffic of people between Pakistan and neighboring countries endemic for tuberculosis such as, migration of populations from Afghanistan, and also travel between neighboring countries including China, Iran, the Middle East, India and Bangladesh. It could however also be due to the presence of hyper variable regions in the strains circulating in this region. Previous studies have suggested that increased strain diversity may also be due to lower transmissibility of indigenous strains [45].

To further understand the genetic character of MTB strains studied, we subjected a subgroup of CAS1 and 'unique' spoligotypes to IS*6110*-RFLP typing. Of the 29 CAS1 strains studied, 27 revealed a variable multi-copy IS*6110*-RFLP profile while two strains had zero copy of IS*6110 *element present. This is the first report of a zero copy IS*6110 *MTB strain as previously CAS1 strains have been shown to have multiple copies of IS*6110 *[46]. One cluster of two strains detected by RFLP typing containing one copy of IS*6110 *was further differentiated by MIRU-VNTR typing, further supporting the higher discriminatory ability of MIRU-VNTR typing especially for low copy IS*6110 *strains [47].

MIRU-VNTR allelic studies have been correlated with definitions of ancestral and modern MTB lineages, with the presence of one allele in locus 24 being related to a modern strain type [18,42]. We found that 62% (107/178) of our CAS1 strains contained only one allele at locus 24, further confirming their modern lineage. This is comparable with previous reports for CAS1 and Beijing strains from Singapore and Bangladesh [18,42] and also from India as supported by the absence of the TbD1 region from their CAS family strains [41].

We also compared our MIRU profiles of the CAS1 family isolates with studies from Russia, Singapore and Bangladesh [18,37,41,42], through an international database [see Additional file 1] and also with CAS strains from India [41]. However, none of the CAS1 MIRU types we identified were shared by those reported previously. This implies that our CAS1 genotypes are generally clonal and corroborates previous work which has suggested this strain family to be a highly diverse genetic group.

We have used the standard 12 loci based method of MIRU-VNTR typing. However, recent studies have identified increasing numbers of related MIRU loci which may help in further discrimination between strains. Supply *et *al. used 29 loci based typing and subsequently recommended 24 loci based typing for phylogentic analysis and 15 loci typing for improved epidemiological studies [48]. They identified MIRUs 10, 26, 40, 31, 4 and 16 as being highly discriminatory (in decreasing order) for routine epidemiological studies [48]. On the other hand Gutierrez *et *al used 21 loci based VNTR typing to study 91 MTB isolates from India [41].

The 12 standard loci analysis we used included all six MIRU loci recommended by Supply et al [48] and also 12 of the 21 loci used by Gutierrez *et *al [41]. While using larger number of loci would certainly be more discriminatory for lineage analysis, our analysis focused more on differentiation within CAS1 strains. As such an overall comparison of MIRU loci for CAS1 and 'unique' strains revealed loci 26, 16, 10, 31, 40, 39, 27, 23, 24, 20, 2 and 4 to be in descending order of discrimination for allelic diversity. Loci 4, 10 and 26 had a significantly lower discriminatory index with a P-value < 0.05 in CAS1 strains than in 'unique', suggesting these loci to be the most conserved in CAS1 strains. In addition, locus 4 of CAS1 MDR strains also had significantly lower discriminatory index with a P-value < 0.05 when compared with MDR 'unique' spoligotype strains. Although, CAS1 strains constituted 53% of the total MDR strains, overall, no significant association of CAS1 family could be established with multidrug resistance.

## Conclusion

The effectiveness of MIRU loci to discriminate between strains may vary between populations. Therefore, it is important to determine the most discriminatory alleles for each country depending on the preponderance of MTB strain types. In a region where CAS1 family of strains are the most prevalent spoligotype we found MIRU loci 26, 31, 16, 10, 27, 39 and 40, in decreasing order, to be the most discriminatory for differentiation of *Mycobacterium tuberculosis*.

## Methods

### Mycobacterial strains

A total of 178 CAS1 strains identified through spoligotyping and 189 'unique' isolates that had been shown to have spoligotype patterns not belonging to any cluster from SpolDB4 [15] were selected from 2003–2005 for this study. These isolates represented different geographical locations across Pakistan were selected through a stratified random sampling method.

### Culture and antibiotic susceptibility testing

All mycobacterial strains were cultured on Middlebrook 7H10 agar. Susceptibilty testing was performed by the standard agar proportion method with enriched Middlebrook 7H10 medium(BBL) as described previously [49-51]. The following final drug concentrations were used: rifampicin, 1 μg/ml and 5 μg/ml; isoniazid, 0.2 μg/ml and 1 μg/ml; streptomycin, 2 μg/ml and 10 μg/ml; ethambutol 5 μg/ml and 10 μg/ml. Pyrazinamide was tested with BACTEC 7H12 medium, pH 6.0, at 100 μg/ml (Becton Dickinson) as per manufacturer's instructions. Strains with a high level of resistance for rifampicin (5 μg/ml) and isoniazid (1 μg/ml) were further selected for MDR analysis.

### MIRU-VNTR PCR

DNA was extracted by cetyltrimethylamonium bromide method [52], twelve MIRU loci (2, 4, 10, 16, 20, 23, 24, 26, 27, 31, 39 and 40) were PCR amplified individually for all 367 isolates using specific primers as described previously [29]. Each of the PCR master mixes contained 0.4 μM concentration of specific primers, 0.5 mM concentration of dNTPs mix, 1 mM concentration of MgCl_2_, 1× PCR buffer, 4 % of DMSO and 1U of Super Tth Taq DNA polymerase for a 25 μl reaction. Master mixes were distributed to 96-well plates. Approximately 40–60 ng of template DNA was added for each sample. *M tuberculosis *H37Rv DNA used as a positive control while negative controls consisting of PCR mixtures lacking mycobacterial DNA was also used. PCR plates were sealed and placed in PerkinElmer 9700 thermocycler starting with a denaturing step of 15 min at 95°C, followed by 35 cycles of 1 min at 94°C, 1 min at 59°C, and 1 min 30 s at 72°C, followed by an extension of 72°C. After the thermocycling step, all 367 MTB isolates were analyzed using a simple gel electrophoresis method. The PCR products were electrophoresed on a 2.5% agarose gel and sized with a 100-bp ladder (Promega). Band sizes were measured using Geldoc Quantity-one (Bio-RAD) soft ware and allelic numbers were determined using the MIRU-VNTR allele scoring table [see Additional file 2].

### IS*6110*-RFLP

IS*6110*-RFLP of 78 *M tuberculosis *strains were performed by standardized methods [53]. Briefly, MTB strains were cultured on Lowenstein-Jensen medium and DNA was extracted from them by standard method [52,53]. *Pvu*II digested DNA was subjected to agarose gel electrophoresis and Southern blotting. DNA fingerprinting was performed by hybridization with the IS*6110 *using enhanced chemiluminescence method (ECL Amersham).

### Phylogenetic Analysis

The twelve digits MIRU-VNTR allele score obtained for each MTB strain was then entered into Bionumerics soft ware (Applied Maths, St. Martens Latem, Belgium) as a character set and used to generate a dendrogram by un-weighted pair group using arithmetic averages (UPGMA). To compare isolates combining both methods, a multi experiment composite data set with MIRU and Spoligotyping was created by using the available tools in Bionumerics.

### Statistical analysis

The Hunter Gaston Discriminatory Index (HGDI) was calculated for comparison of discriminatory power of MIRU-VNTR typing for different loci [54]. Non parametric analysis was carried out using the Mann-Whitney test to determine the utility of MIRU typing to distinguish between CAS1 and 'unique' as well as CAS1-MDR and 'unique' MDR. A P value of < 0.05 was considered significant. This analysis was carried out using version 14 of SPSS (Special Program for Social Sciences Software, USA).

## Authors' contributions

RH supervised the research study. ZH supervised the experimental work and manuscript preparation. MT performed spoligotyping. RS provided statistical support. AA cultured the *M tuberculosis *strains, performed MIRU and IS*6110*-RFLP typing and prepared the manuscript. SG provided technical assistance for RFLP IS*6110 *typing which was supported by GK. All the authors provided feedback in manuscript preparation.

## Supplementary Material

Additional file 1 MIRU database. Twelve loci based MIRU profiles accessed from international database link . Click here for file

Additional file 2 MIRU-VNTR allele scoring table. MIRU-VNTR allele scoring table accessed from international database link  for MIRU typing. Click here for file
