# Supplementary material for: Characterization of Mycobacterium tuberculosis Central Asian Strain1 using mycobacterial interspersed repetitive unit genotyping
Source: BMC Microbiol. 2007 Aug 9;7:76. doi: 10.1186/1471-2180-7-76 (PMC1988810; doi:10.1186/1471-2180-7-76)
Supplement: Additional file 2 — MIRU-VNTR allele scoring table. MIRU-VNTR allele scoring table accessed from international database link for MIRU typing. [file 1471-2180-7-76-S2.RTF]

MIRU-VNTR allele tables
General information
The tables shown below provide the correspondences between the sizes of expected PCR products and the MIRU-VNTR alleles for the 12 MIRU-VNTR polymorphic loci.
Table 1 gives the correspondences using the oligonucleotides reported in Supply et al., 2000, Mol Mic, 36, 762-771 and Mazars et al 2001, PNAS, 98, 1901-1906, describing the genotyping with standard agarose gel electrophoresis; as well as in Supply et al. 2001, J. Clin. Microbiol., 39, 3563-3571, describing the genotyping on fluorescence-based automated sequencers. In the latter study, the oligonucleotides against the regions flanking locus 23 and 26 have been changed to shorten the PCR products. Therefore, these two pairs of new oligonucleotides are also recommended for analysis on agarose gels.
Table 2 displays rare specific alleles in locus 4 (see below).
Using agarose gels versus automated sequencer
MIRU-VNTR genotyping using agarose gels is inexpensive and is accurate, provided that careful electrophoresis conditions are used. We recommend the use of 2% Nu-Sieve agarose (FMC), and 20- and 100-bp superladders-low from Eurogentec, giving the most accurate sizing of PCR products in our hands, which has been validated by sequencing (see Supply et al., 2000, Mol Mic, 36, 762-771; Mazars et al 2001, PNAS, 98, 1901-1906). We strongly recommend to include a H37Rv positive control for amplification and sizing, to validate your own analyses.
MIRU-VNTR genotyping on automated sequencers is more expensive, but much more faster and "user-friendly" with the help of dedicated softwares (Genotyper and Genescan from ABI). The latter method will be preferable when large number of isolates are to be analyzed.
Nomenclature
The MIRU units taken into account are only those which vary in copy numbers (see Supply et al., 2000, Mol Microbiol, 36, 762-771 and references therein for additional information). Loci 2 and 24 contain an additional invariable type III and type I MIRU, respectively. Locus 4 contains an additional invariable 53-bp type II MIRU in 3' of 77-bp VNTR units in nearly all clinical isolates. In Table 2 below, the ' symbol for MIRU numbers in locus 4 designates very rare alleles devoid of 3'-terminal 53 bp MIRU-unit. Such alleles are encountered in BCG strains, H37Rv, H37Ra and in less than 1 % of other Tb strains. Therefore, in our published nomenclature, VNTR numbers in locus 4 designate only the numbers 77-bp units with the presence of a 3' terminal 53-bp unit being implicit (exceptions are mentioned as footnotes or in the text in our papers).
Please notice that for some loci (locus 10 for instance), the first repeat unit in the repeat array may have a different size than the following ones (see Supply et al., 2000, Mol Microbiol, 36, 762-771). This is taken into account in the calculations below.

Table 1: main MIRU-VNTR allele table

Allele	MIRU 02	MIRU 04	MIRU 10	MIRU 16	MIRU 20	MIRU 231	MIRU 232	MIRU 24	MIRU 261	MIRU 262	MIRU 27	MIRU 31	MIRU 39	MIRU 40	
0	402	175	482	565	437	558	150	395	461	285	498	492	540	354	
1	455	252	537	618	514	608	200	447	512	336	551	545	593	408	
2	508	329	590	671	591	661	253	501	563	387	604	598	646	462	
3	561	406	643	724	668	714	306	555	614	438	657	651	699	516	
4	614	483	696	777	745	767	359	609	665	489	710	704	752	570	
5	667	560	749	830	822	820	412	663	716	540	763	757	805	624	
6	720	637	802	883	899	873	465	717	767	591	816	810	858	678	
7	773	714	855	936	976	926	518	771	818	642	869	863	911	732	
8	826	791	908	989	1053	979	571	825	869	693	922	916	964	786	
9	879	868	961	1042	1130	1032	624	879	920	744	975	969	1017	840	
10	932	945	1014	1095	1207	1085	677	933	971	795	1028	1022	1070	894	
11	985	1022	1067	1148	1284	1138	730	987	1022	846	1081	1075	1123	948	
12	1038	1099	1120	1201	1361	1191	783	1041	1073	897	1134	1128	1176	1002	
13	1091	1176	1173	1254	1438	1244	836	1095	1124	948	1187	1181	1229	1056	
14	1144	1253	1226	1307	1515	1297	889	1149	1175	999	1240	1234	1282	1110	
15	1197	1330	1279	1360	1592	1350	942	1203	1226	1050	1293	1287	1335	1164	
1with oligonucleotides used in Supply et al., 2000, Mol. Microbiol., 36, 762-771 and described in Mazars et al 2001, PNAS, 98, 1901-1906 (see supplemental data on the PNAS Web site http://www.pnas.org)
2with oligonucleotides used in Supply et al. 2001, J. Clin. Microbiol., 39, 3563-3571


Table 2: specific MIRU-VNTR alleles in locus 4 (see nomenclature above)

Allele	MIRU 04	
0'	122	
1'	199	
2'	276	
3'	353	
